# Brain Reward and Stress Systems in Addiction

**DOI:** 10.3389/fpsyt.2014.00079

**Published:** 2014-07-09

**Authors:** Nicholas W. Gilpin

**Affiliations:** ^1^Department of Physiology, Louisiana State University Health Sciences Center New Orleans, New Orleans, LA, USA

**Keywords:** relapse, reward, stress, pain, alcohol, nicotine, heroin, methamphetamine

Addiction to drugs and alcohol is a dynamic and multi-faceted disease process in humans, with devastating health and financial consequences for the individual and society at large. The recently released fifth edition of the *Diagnostic and Statistical Manual of Mental Disorders* (DSM-V) combined the previously separate abuse and dependence classifications for licit and illicit drugs of abuse into a single syndrome called substance use disorder (SUD). This new definition includes diagnostic criteria that are largely overlapping with previous criteria (DSM-IV), and new diagnostic thresholds wherein physicians are charged with classifying the severity of an individual’s SUD based on the number of criteria met. More specifically, mild SUD requires that two to three symptoms be met, moderate SUD requires that four to five symptoms be met, and severe SUD requires that six or more symptoms be met. One notable addition to diagnostic criteria is craving, which can be defined broadly as a strong desire or urge to use drug/alcohol. Different classes of abused drugs can have different biological consequences and different co-morbidity risks, but SUDs are defined and diagnosed according to a single set of behavioral symptoms that are common to abuse of all drugs. These behavioral symptoms include compulsive drug use, loss of control in limiting drug intake, the emergence of a negative emotional state in the absence of the drug, and increased vulnerability to relapse triggered by stress or cues previously associated with drug availability. Each of these symptoms can be modeled to various degrees in animals, and animal models are particularly useful for exploring the underlying neurobiology of SUD and for identifying promising new targets for treatments aimed at curbing excessive drug and alcohol use in humans.

The main purpose of this Research Topic is to consolidate review and empirical articles by leaders in the addiction field that collectively explore the contribution of brain reward and stress systems in addiction. The transition to severe SUD is defined by neuroadaptations in brain circuits that, in the absence of drugs, are responsible for mediating behavioral and physiological processes that include motivation, positive and negative emotional states, nociception, and feeding. Chronic drug exposure during this transition promotes (1) within-system changes in neural circuits that contribute to the acute rewarding effects of the drug and (2) recruitment of both hypothalamic (neuroendocrine) and extra-hypothalamic brain stress systems.

Various biological and behavioral processes contribute to the propensity of an individual to use and abuse drugs and alcohol. For example, links are emerging between specific genetic profiles and diagnoses of SUDs. Furthermore, drug and alcohol abuse are highly co-morbid with other psychiatric conditions (e.g., anxiety disorders, major depressive disorder, schizophrenia, and personality disorders) that may precede or follow the development of drug use problems. Across different drugs of abuse, there are overlapping and dissociable aspects of the behavioral and neurobiological changes that define the transition to dependence. Even within a single drug of abuse, different people abuse drugs for various reasons; within a single individual, the reasons for drug abuse may change across the lifespan and the course of the disorder. The picture is further complicated by the fact that humans often abuse more than one drug concurrently.

This Research Topic begins with a review by Dr. George Koob, Ph.D., newly appointed Director of the National Institute on Alcohol Abuse and Alcoholism (NIAAA), that describes addiction as a disorder mediated by pathophysiological reductions in brain reward function and concurrent recruitment of brain stress circuits (1). Several of the articles that follow build on the idea that recruitment of brain stress systems [e.g., corticotropin-releasing factor (CRF) and glucocorticoids] is critical for promoting excessive drug and alcohol use. The remainder of this Research Topic is a collection of empirical and review articles that describe work aimed at unraveling the neurobiology of addiction to various drugs of abuse, and that ties this neurobiology with various current “hot topics” in the addiction research field (2–14).

The articles in this Research Topic address various points of current emphasis in the addiction research field. One such area is the idea of individual differences: it is gradually being accepted that addicts across and within drugs of abuse are not all the same, that individuals may arrive at the same phenotypic or diagnostic endpoint by different life paths and precipitating factors, that individuals exhibit different sets of co-morbidities (e.g., addiction and pain), and that therapeutic approaches and clinical trials may be more effective if tailored to subpopulations of addicts (i.e., pharmacogenetics). Also addressed in this set of articles is the notion that individual neurochemical systems may be critical for mediating not only abuse of more than one drug, but for mediating co-abuse of more than one drug in a single individual (e.g., the high rates of co-morbid smoking in individuals with alcohol use disorder). Another area of major social concern that is currently receiving much attention in the addiction research field is the drive to understand the long-term effects of adolescent drug and alcohol exposure on brain and behavior. It is generally accepted that early initiation of drug and alcohol use increases the risk for development of SUD and other psychiatric conditions later in life, and this may be due to the fact that the adolescent brain, because it is still developing, is particularly vulnerable to the effects of these substances.

Pre-clinical research utilizes a variety of animal models and rapidly advancing technological approaches to explore the underlying neurobiology of drug addiction. Several articles in this Research Topic describe commonly used genetic models (e.g., selective breeding animals for high alcohol preference) and more recently developed exposure models (e.g., nicotine vapor as a model for e-cigarettes and second-hand smoke) of addiction. These models can be combined with new technologies (e.g., optogenetics and chemogenetics) to examine the neurobiology of addiction in increasingly sophisticated ways, for example, the approach of isolating single brain regions is quickly being replaced by circuitry approaches, and intra-cranial delivery of drug solutions with “dirty” receptor binding and diffusion profiles are being replaced by highly controllable optical stimulation and designer drug techniques. Collectively, the articles presented here provide a snapshot of the current theoretical and experimental landscape in the addiction research field.

## Conflict of Interest Statement

The author declares that the research was conducted in the absence of any commercial or financial relationships that could be construed as a potential conflict of interest.
